# Delivery strategies for RNA-targeting therapeutic nucleic acids and RNA-based vaccines against respiratory RNA viruses: IAV, SARS-CoV-2, RSV

**DOI:** 10.1016/j.omtn.2025.102572

**Published:** 2025-05-20

**Authors:** Kinga Maziec, Agnieszka Baliga-Gil, Elzbieta Kierzek

**Affiliations:** 1 Institute of Bioorganic Chemistry, Polish Academy of Sciences, Noskowskiego 12/14, 61-704 Poznan, Poland

**Keywords:** MT: Delivery Strategies, delivery methods, RNA therapeutics, ASO, oligonucleotides, mRNA vaccine, influenza A, SARS-CoV-2, RSV

## Abstract

Therapeutic nucleic acids, including small interfering RNA (siRNA), and antisense oligonucleotides (ASOs), targeting RNA viruses such as influenza A virus (IAV), severe acute respiratory syndrome coronavirus 2 (SARS-CoV-2), and respiratory syncytial virus (RSV), play a crucial role in contemporary medicine. The primary goal of short oligonucleotide-based antivirals is to precisely inhibit viral mechanisms by interacting with viral RNA, thereby opening new avenues for infection treatment. RNA recently was also used to invent mRNA vaccine for different illness prevention. Therapeutic nucleic acids and mRNA vaccine attracted considerable attention during the COVID-19 pandemic due to the pressing necessity to develop an effective strategy to address this global threat. In addition to the advancement of therapeutic nucleic acids aimed at targeting respiratory viruses, the effective delivery of these molecules to infected cells is of paramount importance. Similarly, mRNA vaccine’s effectiveness also depends on effective delivery. This article offers a comprehensive summary and analysis of various delivery strategies, along with the challenges encountered in their development. Representative studies conducted in cellular models, model organisms, and human are presented for examination. Furthermore, the article explores future perspectives regarding the delivery of therapeutic nucleic acids and mRNA vaccines aimed at combating IAV, SARS-CoV-2, and RSV.

## Graphical abstract

**Figure:**
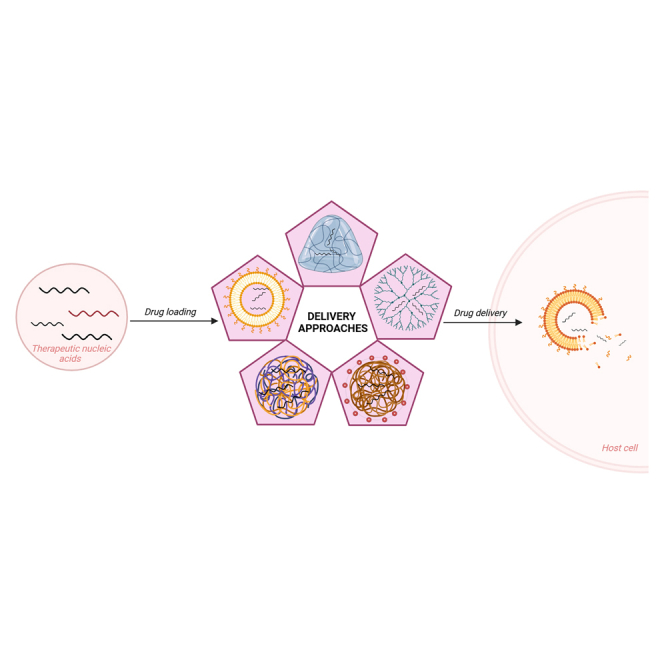

## Teaser

This publication addresses therapeutic nucleic acid delivery methods and mRNA vaccines that target respiratory viruses. The paper discusses the transport strategies, efficacy, and challenges of these approaches. The importance of the methods described in the context of modern antiviral therapies and infection prevention is also highlighted.

## Introduction

**Figure 1:**
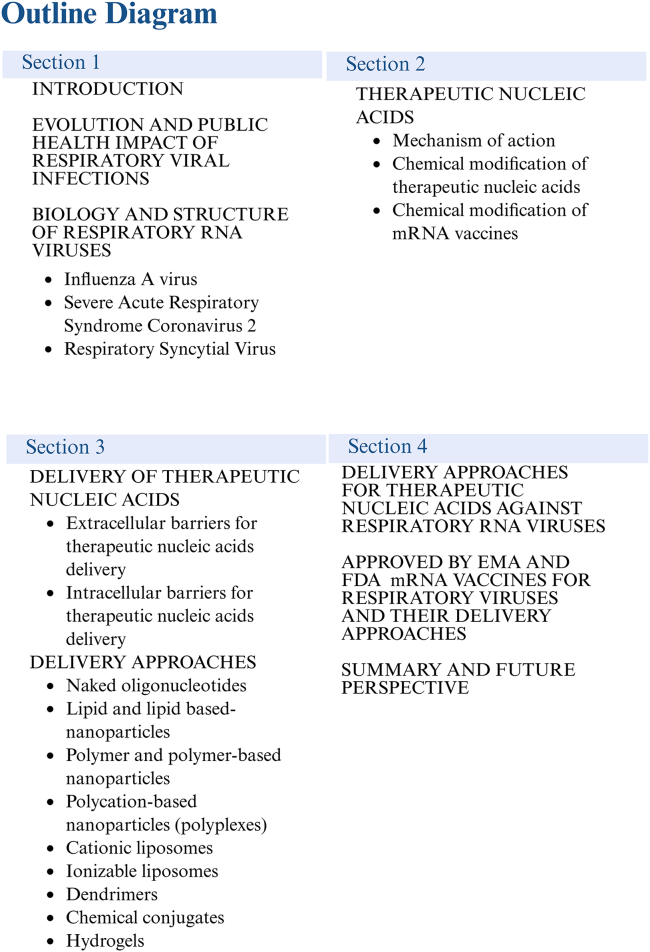
Outline diagram visualizing the structure of the manuscript

The essential role of RNA was underscored by the discovery of mRNA, which serves as a pivotal component in the process of translation. This discovery also revealed RNA’s capacity for self-hybridization, leading to the formation of A-form helices, as well as various two-dimensional and complex three-dimensional structural motifs. These significant findings established the groundwork for subsequent advancements, including the elucidation of the correlation between RNA structure and function, the identification of non-coding regulatory RNAs such as microRNAs (miRNAs), and the phenomenon of RNA silencing.^1^ Given its fundamental role in the regulation of protein expression and various biological processes, RNA has garnered increased attention for its therapeutic applications in the treatment of human diseases (Figure 1).^1^ Initially, both RNA and DNA oligonucleotides were regarded as unsuitable therapeutic nucleic acids due to their limited stability *in vivo*. However, recent advancements in chemical modification techniques have significantly improved their stability, thereby alleviating concerns regarding their application in disease treatment. The most common chemical modifications that stabilize oligonucleotides are locked nucleic acid (LNA), peptide nucleic acid (PNA), and phosphorodiamidate morpholino oligomer (PMO).^2^^,^^3^^,^^4^ In recent years, research has concentrated on the therapeutic potential of RNA, culminating in the development of antisense oligonucleotides (ASOs) that utilize chemically modified RNA and siRNAs, which are now being employed clinically. Moreover, extensive efforts are currently underway to create RNA-based therapeutic formulations, including RNA aptamers, and mRNA therapies and vaccines.^2^^,^^3^^,^^4^ Viruses whose genetic material is RNA have become an object of interest due to their prevalence and variability. RNA, as a genetic material, is prone to rapid mutation, which contributes to the emergence of new viral variants, which in turn leads to difficulties in developing effective therapies. At the same time, viral RNA genomics is undergoing development, representing increasingly important roles in the clinical studies.^5^^,^^6^^,^^7^^,^^8^

## Evolution and public health impact of respiratory viral infections

Most viral respiratory infections are caused by RNA viruses, which rapidly spread through airborne aerosols or direct contact with an infected individual. This mode of transmission facilitates efficient viral dissemination, leading to seasonal epidemics and, in more severe cases, pandemics characterized by heightened morbidity and mortality. The ability of these viruses to cross species barriers drives genetic variations, ultimately resulting in the emergence of new variants and strains with enhanced infectivity. To date, several technologies leveraging the therapeutic potential of nucleic acids have been investigated and implemented to combat respiratory system infections caused by these viruses. Research has shown the efficacy of some of these methods in clinical trials aimed at addressing influenza A virus (IAV), severe acute respiratory syndrome coronavirus 2 (SARS-CoV-2), and respiratory syncytial virus (RSV). The efficacy of the therapeutic nucleic acids was assessed *in vivo* by measuring viral titers, utilizing qPCR to analyze viral RNA (vRNA) levels, evaluating viral protein expression via Western blot, and examining histopathological changes in infected tissues.^9^^,^^10^^,^^11^^,^^12^

Respiratory infections on a global scale are most often caused by IAV.^13^ IAV infection results in recurrent seasonal epidemics and occasional pandemics, with seasonal influenza being driven by the emergence of new viral strains that exhibit antigenic differences from previous epidemic variants. These novel antigens arise due to the accumulation of mutations introduced by errors in vRNA replication, a process mediated by RNA-dependent RNA polymerase (RdRp) and commonly referred to as “antigenic drift.”^14^ Another fundamental and critical mechanism in the evolution of IAV is known as “antigenic shift.” This process involves the reassortment of viral RNA (vRNA) segments between two distinct IAV strains during coinfection.^15^ The best known and deadliest viral influenza pandemic was the “Spanish flu” pandemic of 1918–1920, which is estimated to have caused around 50 million deaths worldwide.^16^ It was hypothesized that the “Spanish flu” pandemic occurred in three waves, but this pattern was not typical for all regions, as in some regions, influenza persisted or declined and returned in 1920. Morbidity and mortality statistics may be significantly underestimated due to probable misdiagnosis or changeability by location.^17^ The last pandemic linked to the IAV started in 2009 in La Gloria, Mexico. It was the result of an IAV (H1N1) virus that was distinguished by an unusual combination of genetic segments from IAV viruses infecting humans and those infecting birds and pigs.^18^

Due to the presence of genetic variability in RNA viruses, including antigenic drift and shift, in IAV, these viruses are able to evade the immune response. Therefore, there is an ongoing need for a universal vaccine that provides protection against IAV infection.^19^ Due to the influenza virus’s ease of mutation, new vaccines must be produced every year. However, research based on structural biology and genetics to determine the immunogenicity and antigenicity of IAV is underway, with the goal of developing a vaccine that will protect against multiple strains.^20^

In late 2019, an increasing number of pneumonia cases were reported in Wuhan, China, triggering a pandemic involving SARS-CoV-2.^21^ On December 31, 2023, the number of people infected with the virus was more than 770 million, and the number of deaths was more than 7 million (data from 27 Apr. 2025, WHO). The symptoms of SARS-CoV-2 and IAV are known to be very similar and include fever, cough, pneumonia, and acute respiratory syndrome. Moreover, SARS-CoV-2 and IAV are airborne pathogens that target the same human tissues, including the airway, nasal, bronchial, and alveolar epithelial cells. Although the symptoms associated with these viruses are similar, their severity and frequency vary. Fever, diarrhea, vomiting, muscle pain, body aches, and chest pain were significantly more prevalent in children with influenza, whereas cough and shortness of breath occurred with equal frequency in infections caused by both viruses. However, adults infected with SARS-CoV-2 experienced higher levels of fatigue, unconsciousness, and diarrhea compared to those with influenza. Moreover, the SARS-CoV-2 virus has the potential to damage various organs, including the thyroid, brain, heart, skeletal muscles, lymph nodes, and the digestive system.^22^^,^^23^ The commitment to develop a vaccine against SARS-CoV-2 came too late to be able to slow or stop the first wave of the COVID-19 pandemic.^24^ However, continuous research to develop vaccines against SARS-CoV-2 has led to the development of effective mRNA-based vaccines. This is due to its ability to induce a strong acquired immune response by providing genetic information for antigen production.^25^ To date, several antibodies directed against different domains of this virus have been identified. There is potential for the use of these antibodies in patient therapy and future vaccines. Significant efforts are currently underway to develop effective and safe therapies and new vaccines against SARS-CoV-2. Various types of vaccines are being tested in clinical trials, including inactivated, nucleic acid-based, and vector-based vaccines. Some of those vaccines (Pfizer/BioNTech’s BNT162b2 and Moderna’s mRNA-1273) have already been approved by the Food and Drug Administration (FDA) and European Medicines Agency (EMA).^26^^,^^27^^,^^28^

Another virus that affects the respiratory system is RSV, which was first identified in 1956. It predominantly affects infants and young children between the ages of 6 weeks and 2 years, typically causing symptoms resembling those of the common cold. The clinical manifestations of RSV infection vary depending on the age of the affected individual. In older children, the disease tends to present with milder symptoms, such as pneumonia, wheezing, and otitis media, and is generally less severe compared to a primary RSV infection.^29^ Many cases of RSV infection occur in children under the age of 1 year. The virus is known to infect people of all ages, but the most severe symptoms appear in the infants, elderly, and those with weakened immune systems.^30^ Despite the challenges associated with developing an effective vaccine, its recent invention has been officially announced. The vaccine was approved by the EMA^31^ and the FDA in 2023 and is intended for infants up to 2 years of age and people 60 years old and older.^32^

To date, several technologies leveraging the therapeutic potential of nucleic acids have been investigated and implemented to combat respiratory system infections caused by these viruses. Frequent epidemics and occasional pandemics caused by respiratory viruses have driven intensified research efforts to combat these pathogens, including the development of RNA-based technologies. Understanding the sequence and structure of these viral genomes is particularly valuable for devising effective disease prevention and treatment strategies. Among the various therapeutic approaches, the use of nucleic acids continues to evolve alongside advancements in their delivery methods. This review provides a comprehensive overview of delivery strategies and modifications of therapeutic nucleic acids targeting RNA, as well as mRNA vaccines developed against respiratory viruses, including IAV, SARS-CoV-2, and RSV (Figure 1).

## Biology and structure of respiratory RNA viruses

### Influenza A virus

**Figure 2:**
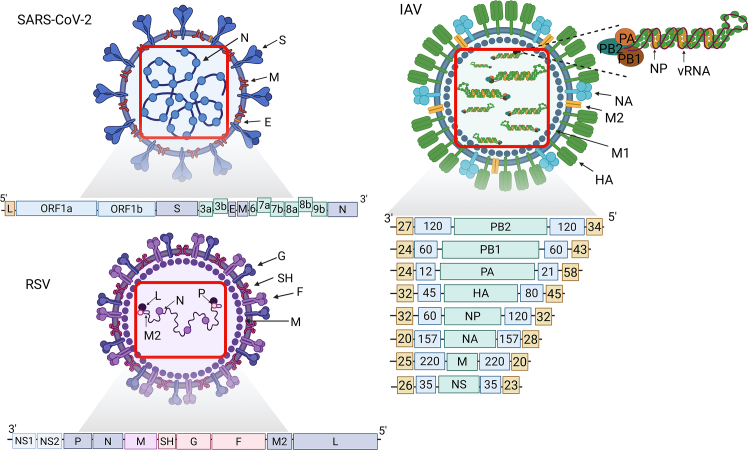
Schematic representation of SARS-CoV-2, IAV, and RSV, along with their genomes The virions structure includes each virus’s main structural proteins, whereas the genome diagrams show their organization and key functional elements. In the IAV genome, the orange boxes at the ends of each vRNA segments represent the 5′ and 3′ untranslated regions (UTRs); the blue boxes represent the packaging signals in the coding region. The numbers inside the boxes represent the nucleotide length for the UTRs and the packaging signals. These values are based on a reference model.

IAV is an enveloped virus belonging to the *Orthomyxoviridae* family. During viral replication, its outer lipid envelope is derived from the host cell membrane. This envelope features spike-like structures, the majority of which are hemagglutinin (HA) proteins. In addition to these spikes, the envelope contains structures resembling fungal projections, composed of neuraminidase (NA) and the matrix protein 2 (M2). Beneath the lipid envelope, the matrix protein 1 (M1) provides structural support and facilitates the attachment of ribonucleoprotein (RNP) complexes. Within the RNP, the nucleoprotein (NP) encapsulates the viral genome, which is associated with a single copy of RNA-dependent RNA polymerase (RdRp), composed of three protein subunits: polymerase acidic protein (PA), polymerase basic protein 1 (PB1), and polymerase basic protein 2 (PB2) (Figure 2).^33^

The genetic material of IAV is ssRNA—divided into eight segments. The fragments are numbered from longest to shortest and are named after the proteins they encode: PB2, PB1, PA, HA, NP, NA, M, and non-structural protein (NSP) (Figure 2). These proteins are encoded by segments in the order of 1–8. IAV can be divided into antigenic subtypes based on the surface glycoproteins HA (classes H1-H18) and NA (classes NA1-NA11). The structural organization of all vRNAs is uniform. Specifically, the open reading frame (ORF), which encodes one or more proteins in the antisense direction, is centrally positioned and flanked by two short untranslated regions (UTRs).^34^^,^^35^ In addition, a packaging signal is present in each of the eight segments as a specific sequence of nucleotides due to which each segment is precisely recognized and incorporated into the forming virion, ensuring the completeness of the genetic material in the progeny virions. The structure of RNA has been demonstrated to play a crucial role in the life cycle of the IAV. Numerous processes occurring throughout the viral replication cycle have been proposed to be dependent on RNA structure, including splicing, nuclear export, translation, and vRNA packaging. Additionally, RNA structure has been implicated in immune system recognition and the regulation of the switch between transcription and replication.^36^ Experimental research was conducted under both *in vivo* and *in vitro* conditions, leading to computational predictions of the IAV RNA genome and mRNA structure. The results highlight the significant role of these structures in essential viral functions.^37^^,^^38^^,^^39^^,^^40^^,^^41^

### Severe acute respiratory syndrome coronavirus 2

Coronaviruses are enveloped viruses classified within the family *Coronaviridae* and the subfamily *Orthocoronavirinae*, among which there are four genera*: alphacoronaviruses, betacoronaviruses, gammacoronaviruses,* and *deltacoronaviruses*. SARS-CoV-2 belongs to the *betacoronaviruses*.^42^ The SARS-CoV-2 virion is ellipsoidal or spherical in shape and consists of the following structural proteins: spike protein (S), nucleocapsid protein (N), membrane protein (M), and envelope protein (E) (Figure 2). Due to the flexible structure of the S proteins present on the viral surface, the virus can effectively explore the host cell surface in search of the angiotensin-converting enzyme 2 (ACE2) receptor. During biosynthesis in infected host cells, the S protein is cleaved into two subunits: S1, which binds to ACE2, and S2, which facilitates membrane attachment. Inside the virion, RNP complexes are present, consisting of the N protein and vRNA.^43^^,^^44^

The SARS-CoV-2 genome is ssRNA+ with a length of approximately 30 kb, making it the virus with the largest genome within the *Orthocoronavirinae* subfamily. It encodes 29 proteins and contains 14 ORFs within its structure. Additionally, the genome features a 5′ cap structure and a poly(A) tail at the 3′ end. Approximately two-thirds of the viral genome encodes two overlapping polyproteins, ORF1a and ORF1ab, which are subsequently processed into 16 NSPs. These NSPs play a crucial role in the replication of genomic RNA and the transcription of subgenomic mRNA. The remaining portion of the genome primarily encodes the previously mentioned structural proteins: S, E, M, and N. To date, many scientific papers have been published on the secondary structure of SARS-CoV-2 RNA and its relevance in the life cycle of the virus. Notably, the secondary structures of the 5′ and 3′ UTRs are critical to the viral replication cycle. The 5′UTR region contains several highly conserved hairpin structures, designated as stem-loop 1 (SL1), SL2, SL3, SL4, SL4.5, and SL5.^45^ The 3′ UTR region, on the other hand, has such motifs as bulged stem-loop (BSL), SL1, highly variable region (HVR), and stem-loop II-like motif (s2m). The 5′ UTR conserved hairpin structures were confirmed by whole-genome mapping, *in vitro* and *in vivo*. In addition, this virus produces subgenomic RNA (sgRNA) as part of its replication cycle. The sgRNA is generated by processes of interrupted transcription and enables more efficient translation and selective expression of structural and regulatory proteins without the need for full genome replication.^46^^,^^47^^,^^48^^,^^49^^,^^50^

### Respiratory syncytial virus

RSV is classified within the genus *Pneumovirus*, subfamily *Pneumoviridae*, family *Paramyxoviridae*, and order *Mononegavirales*. RSV exists in two antigenic subtypes, A and B, which exhibit sequence variations across the genome. RSV is an enveloped virus that adopts either a spherical or an elongated filamentous morphology under *in vitro* production conditions. Its lipid envelope comprises three distinct types of surface transmembrane proteins: glycoprotein (G), fusion protein (F), and a small hydrophobic protein (SH). Viral glycoproteins form separate structures composed of identical units (homooligomers), which manifest as short spikes on the surface of the envelope. On the inner side of the envelope is a non-glycosylated matrix protein (M) (Figure 2). Due to the absence of hemagglutinin and neuraminidase, the F protein is highly sialylated, likely as a consequence of the lack of neuraminidase. The RSV ribonucleocapsid consists of four proteins: the nucleoprotein (N), the phosphoprotein (P), the transcription processivity factor (M2-1), and the large polymerase subunit (L).^51^^,^^52^

RSV is a virus whose genetic material is not segmented ssRNA.^53^ In the organization of the RSV genome, 11 ORFs encoding 9 structural proteins and 2 non-structural proteins (NS1, NS2) can be distinguished^54^; in addition, an ORF encoding the M2 protein (M2-1) is superimposed on the ORF encoding the M2-2 protein.^55^ In addition, learning about the unique nature of RSV polymerase has made it an excellent target for antiviral therapy.^56^ The molecular structure, functions, and mechanisms of RSV *in vitro* have gained attention, contributing to the development of new inhibitors targeting stages of the RSV replication cycle.^57^^,^^58^ The RSV virion structure presented in Figure 2 includes each virus’s main structural proteins, whereas the genome diagrams show their organization and key functional elements.

## Therapeutic nucleic acids

Therapeutic nucleic acids are short strands of nucleic acids and can be used in various therapeutic methods. Due to their mechanism of action, types of DNA or RNA molecules are divided into ASOs, siRNAs, and aptamers. Therapeutic nucleic acids interact with their targets through Watson–Crick complementary base pairing, resulting in degradation by RNase H or inducing steric hindrance that disrupts essential biomolecular interactions. Therapeutic nucleic acids provide several advantages, including precise gene expression inhibition, high versatility, and minimal cytotoxicity. However, one of the major challenges in using ASOs and siRNAs against RNA viruses is the potential for viral escape due to high mutation rates. Genetic variability can lead to mutations in target sequences, reducing the binding efficiency of therapeutic nucleic acids and diminishing therapeutic effectiveness. Strategies such as targeting highly conserved genomic regions, using combinations of multiple therapeutic nucleic acids, and employing chemically modified nucleotides to enhance stability and specificity have been proposed to counteract this. Despite the challenges, oligonucleotides have become valuable tools in antiviral therapy, demonstrating effectiveness against viral infections.^59^^,^^60^

### Mechanism of action

**Figure 3:**
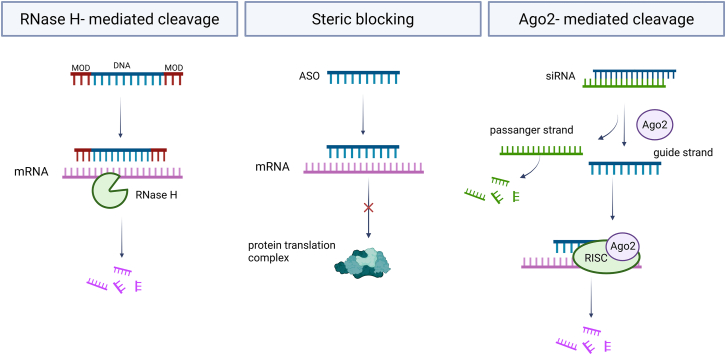
Molecular mechanisms of action of ASO and siRNA on the example of mRNA as a target

Considering the mode of action, they are categorized as either RNA-cleaving/degrading or RNA-blocking/steric-hindrance therapeutic nucleic acids (Figure 3).^61^ The degradation of target RNA by ASO involves the activation of RNase H. This process involves forming a duplex of the target RNA and complementary ASO that must contain a DNA core. Subsequently, the RNase H enzyme is recruited, initiating the degradation of the RNA within the RNA/DNA heteroduplex. Consequently, when targeting mRNA, the translation is hindered, leading to a reduction in the expression of the corresponding protein.^62^ Furthermore, ASOs that do not induce degradation serve as effective methods for regulating gene expression. These therapeutic nucleic acids function as steric blockers, interfering with the splicing machinery, protein-RNA interactions, and RNA secondary structures.^63^ In contrast, siRNA activates the Ago2 protein, which is the only protein directed by siRNA capable of acting as an RNA endonuclease that cleaves a phosphodiester bond in the target mRNA, causing its knockdown.^64^

### Chemical modification of therapeutic nucleic acids

**Figure 4:**
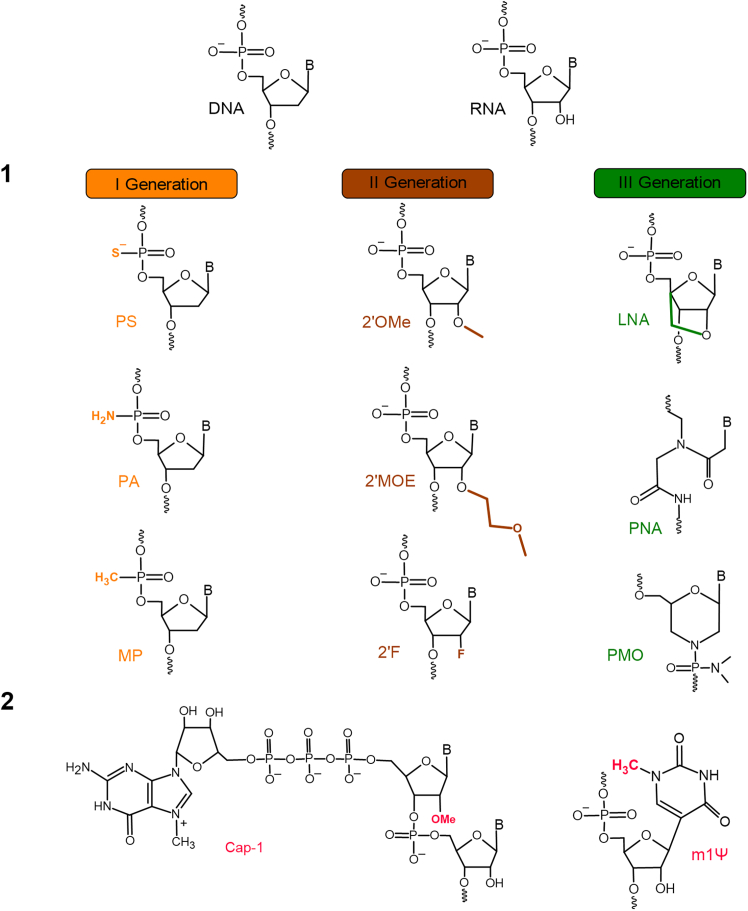
Generations of antisense oligonucleotides. (1) The chemical structure of the first, second, and third generations of oligonucleotides, compared to DNA and RNA structures. DNA, deoxyribonucleic acid; RNA, ribonucleic acid; oligonucleotides modifications: PS, phosphorothioate oligonucleotide; PA, phosphoramidate oligonucleotide; MP, methylphosphonate oligonucleotide; 2′-MOE, 2′-O-methoxyethyl RNA; 2′-OMe, 2′-O-methyl RNA; 2′F, 2′-fluoro RNA; LNA, locked nucleic acid; PNA, peptide nucleic acid; PMO, phosphorodiamidate morpholino oligonucleotide. (2) Chemical structure of modifications used in RNA vaccines. Cap-1, Cap structure on 5′ end of mRNA: m7GpppNm, where Nm is a 2′ -methylated nucleotide; m1Ψ, N1-methylpseudouridine.

Direct chemical modifications have been designed to overcome biological barriers, enabling the safe delivery of therapeutic nucleic acids to their target sites while also enhancing their binding properties. One of the primary biological barriers that these modifications address is the rapid degradation of unmodified oligonucleotides by nucleases in biological fluids, which significantly limits their stability and efficacy. Additionally, negatively charged oligonucleotides face challenges crossing the hydrophobic cell membrane, further restricting their intracellular availability. There are several generations of modifications, depending on their locations. The first generation is characterized by the replacement of the oxygen atoms of the phosphodiester bond with a sulfur (PS), amide (PA), or methyl (MP) group (Figure 4). These modifications improve membrane penetration of therapeutic nucleic acids, do not interfere with RNA cleavage by RNase H, and impart resistance to endonucleases.^65^^,^^66^ The second generation includes modifications of ribose at the 2′-O position of RNA and 2′ position of DNA, incorporating modifications such as 2′-O-methyl (2′-OMe), 2′-O-methoxy-ethyl (2′-MOE), and 2′-fluoro (2′-F) (Figure 3).^62^ The second-generation oligonucleotides have a relatively high affinity for the target. Moreover, the mechanism of action of these modified oligonucleotides leads to steric blockage of translation.^67^ The third-generation oligonucleotides display higher heterogeneity due to incorporation of numerous modifications designed to enhance binding affinity and nuclease resistance, optimizing pharmacokinetics and biostability.^68^ The three most extensively studied are LNA, PNA, and PMO (Figure 4).^3^ The bicyclic sugar modification in LNA effectively locks its conformation, leading to a significant increase in the binding affinity of ASO. Accordingly, LNAs facilitate high-affinity base pairing with complementary target nucleic acids and confer nuclease resistance.^69^ Apart from the primary variants of modified oligonucleotides, many laboratories are working on the next generation of nucleotide modifications. Modifications of oligonucleotides, in addition to changing their binding and resistance properties, are also a way to increase their uptake and are used as a delivery option, thereby overcoming both enzymatic degradation and limited cellular uptake—the two key biological barriers to effective delivery of therapeutic nucleic acids.

### Chemical modification of mRNA vaccines

Naturally, cells have developed receptors that are capable of recognizing the threat of viral infection. When this happens, these receptors trigger defense mechanisms.^70^ A study by Kariko et al. showed that natural RNA modifications, such as pseudouridine, thiouridine, and 5-methylcytidine, reduce mRNA immunogenicity (Figure 4).^71^ This initiated intensive research, and nucleotide base modifications were shown to enhance protein production from synthetic mRNA molecules. Further research led to work on N1-methylpseudouridine (m1Ψ) modification in mRNA, which found application in the development of vaccines against SARS-CoV-2.^72^^,^^73^ The use of m1Ψ in mRNA vaccines is designed to reduce immunogenicity and increase the production of the protein encoded by the mRNA. This allows these vaccines to take advantage of the natural mechanism of mRNA translation while minimizing the risk of adverse side effects such as anaphylaxis.^70^^,^^74^

Modifications to the cap structure (CAP) of mRNA increase the stability of the molecule and the efficiency of translation by favorably affecting the affinity of mRNA for ribosomes (Figure 4).^75^ Modifications of the 5′ CAP are also present in Pfizer and Moderna vaccines. A synthetic trinucleotide CAP analog was used to create the mRNA transcripts present in Pfizer’s vaccine. In contrast, mRNA transcripts containing the CAP-1 structure (m7GpppNm, where Nm is a 2′ -methylated nucleotide),^76^ present in Moderna’s vaccine, were created by enzymatic action. The enzyme used was Vaccinia capping enzyme and Vaccinia 2-O-methyltransferase.^76^^,^^77^^,^^78^

Recent advancements in the field of mRNA modification strategies have highlighted the critical importance of UTRs as well as the 3′ poly(A) tail in enhancing mRNA stability, translational efficiency, and immunogenicity. Both the 5′ and 3′ UTRs play a significant role in the recruitment of ribosomes and the regulation of translation. Research has demonstrated that mRNA possessing minimalist UTR regions can maintain high functionality under both *in vitro* and *in vivo* conditions, suggesting that these regions may be designed to optimize translation while simultaneously minimizing the activation of immune responses.^79^ Furthermore, modifications to the poly(A) tail, including alterations in both length and chemical composition, enhance mRNA stability and protein synthesis by facilitating interactions with poly(A)-binding proteins (PABP) and translation initiation factors.^80^ It is also essential to emphasize the significance of research on mRNA-based vaccines that have examined the effects of modifications to the 3′ UTR and poly(A) tail on the prolongation of mRNA half-life and the improvement of translational efficiency, which have proven vital in the context of developing a new generation of mRNA-based vaccines.^81^ These findings underscore the importance of refining not only coding sequences but also regulatory elements of mRNA to maximize its stability, efficacy, and safety in clinical applications.

## Delivery of therapeutic nucleic acids

Since RNA-based therapeutics have been gaining popularity, developing effective strategies for their delivery to cells is essential in their application.^82^ The developed therapeutic nucleic acids must overcome barriers related to their delivery. The concept of the endosomal escape barrier is now widely regarded as perhaps the most important obstacle to the successful use of oligonucleotides as therapeutics. In addition, therapeutics must avoid lysosomal degradation and entrapment in secretory vesicles which contents are exported from cells. Another difficulty is the possible degradation of oligonucleotides by RNases. In addition, targeting therapy is quite a challenge to ensure the effectiveness of treatment/prevention. Due to the high demand, methods of delivering and modifying oligonucleotides are constantly being improved.^83^^,^^84^^,^^85^ Obstacles to the delivery of therapeutic nucleic acids can be categorized as those of extracellular and intracellular origin.

### Extracellular barriers for therapeutic nucleic acid delivery

For the effective and efficient delivery of therapeutic nucleic acids, it is essential to first overcome extracellular barriers, thereby minimizing the presence of therapeutics in non-target cells. Additionally, administered oligonucleotides may induce an undesired immune response or interact with blood components, potentially affecting their therapeutic efficacy and safety.^86^^,^^87^ Although proteins can bind on the surface of nanoparticles used for delivery, this can lead to their recognition and removal by macrophages, facilitating the recognition and subsequent clearance of nanoparticles from the bloodstream.^88^ This process does not necessarily pose a barrier to effective delivery. In fact, the biomolecular corona of nanoparticles, which includes their interactions with plasma proteins, may play a crucial role in recognition by target cells, such as hepatocytes, thereby enhancing the effectiveness of delivery. Various modifications can be applied to nanoparticles to enhance the biodistribution of delivery systems and the therapeutic nucleic acids they transport. Alterations in surface properties, size, or surface charge may effectively reduce macrophage-mediated clearance, thereby improving the stability and efficiency of nanoparticle-based delivery.^89^^,^^90^

### Intracellular barriers for therapeutic nucleic acid delivery

An additional challenge for therapeutic applications is the presence of intracellular barriers. After traversing the cell membrane, therapeutic nucleic acids are internalized via endocytosis. To exert their therapeutic effect, therapeutic nucleic acids must efficiently escape the endosomal vesicle and reach the target cellular compartment. However, as these vesicles undergo maturation, both the delivery carrier and the nucleic acid cargo are subjected to degradation due to the increasingly acidic environment and the activity of lysosomal hydrolases.^91^ Beyond enzymatic degradation, several other intracellular obstacles must be overcome to ensure the effective delivery of therapeutic nucleic acids. These additional barriers include cellular excretion through exocytosis or recycling pathways, as well as the efficiency of vector unpacking and the decomplexation of nucleic acids, all of which significantly impact therapeutic efficacy,^92^^,^^93^ as well as the potential for the removal of the resulting endosomes and their cargo through the process of autophagy. The nuclear envelope presents an additional challenge for therapeutics requiring delivery to the cell nucleus. Understanding the intracellular barrier is an aspect of developing effective delivery methods. Physical techniques such as electroporation, photoporation, and sonoporation are used to overcome these barriers. The visualization of interactions between delivered therapeutic system (or their carriers) and intracellular barriers also can be valuable, but it requires a precise and advanced methodological approach.^94^ The aforementioned barriers necessitate the development, refinement, and optimization of therapeutics delivery methods, thereby enhancing the efficacy of therapies based on nucleic acids, particularly in cases where the target cells have a limited capacity to accommodate nucleic acids.^95^^,^^96^

## Delivery approaches

**Figure 5:**
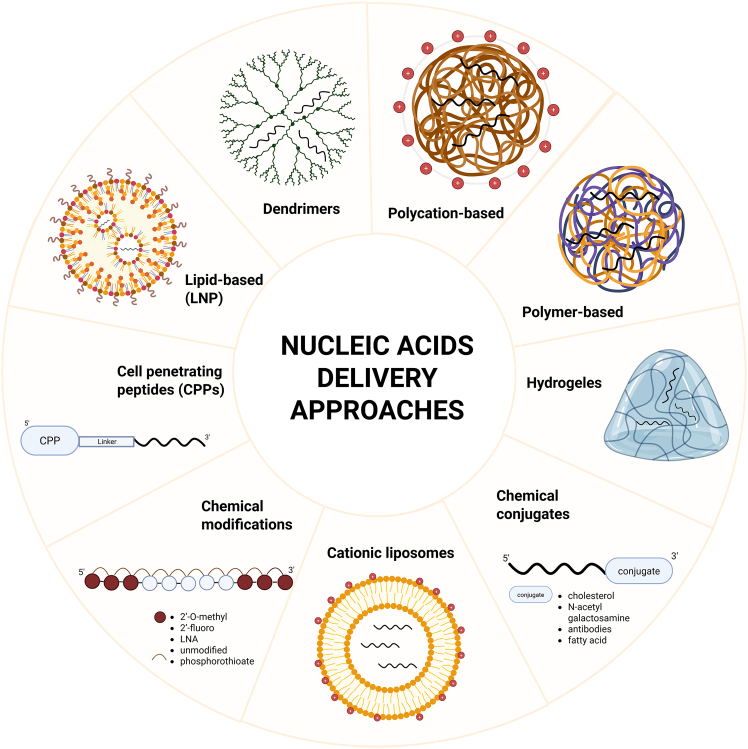
Scheme of delivery technologies for nucleic acids

Oligonucleotide delivery methods can be divided into two broad groups. The first involves the introduction of specific chemical modifications, such as targeting ligands, to oligonucleotides in a manner that preserves or enhances their functionality. The second approach entails the incorporation or encapsulation of oligonucleotides within carriers of various forms, facilitating their effective transport and delivery.^85^ Different nucleic acid cargoes engage in distinct biochemical mechanisms. However, their effective therapeutic application requires precise delivery to the appropriate tissues and cell types. Additionally, therapeutic nucleic acids must not elicit an undesirable immune response or be prematurely cleared by the target organs.^97^ The following section will outline the various therapeutic nucleic acid delivery methods, as well as mRNA-based vaccine delivery technologies (Figure 5; Tables 1 and 2).

**Table 1:** Overview of the advantages and disadvantages of various delivery methods to cells

Delivery approaches	Advantage	Disadvantage
Naked oligonucleotides	✔ ease of synthesis ✔ possibility of chemical modifications ✔ higher efficacy with local delivery ✔ potential for reduced dosing frequency	✖ poor membrane penetration ✖ frequent administration required ✖ instability in the bloodstream ✖ potential interactions with serum proteins
Lipid and lipid-based nanoparticles	✔ high biocompatibility ✔ efficient encapsulation ✔ enhanced endosomal escape ✔ controlled and targeted delivery ✔ ease of modification ✔ structural flexibility	✖ complex production process ✖ temperature sensitivity ✖ potential stability and solubility issues
Polymer and polymer-based nanoparticles	✔ structural versatility ✔ efficient encapsulation ✔ enhanced endosomal escape ✔ controlled drug release ✔ improved bioavailability ✔ protection against degradation	✖ potential toxicity ✖ complex and costly production ✖ physicochemical instability
Polycation-based nanoparticles (polyplexes)	✔ multifunctionality ✔ protection against nuclease degradation ✔ enhanced cellular uptake ✔ improved endosomal escape ✔ surface modification potential	✖ potential immune response ✖ suboptimal delivery efficiency ✖ potential toxicity
Cationic liposomes	✔ efficient encapsulation ✔ ease of production ✔ low immunogenicity ✔ high transfection efficiency ✔ capacity for large therapeutic loads	✖ serum protein interactions ✖ reduced *in vivo* efficacy ✖ potential toxicity
Ionizable liposomes	✔ high biocompatibility ✔ efficient drug release ✔ versatile delivery system ✔ reduced risk of adverse effects	✖ rapid clearance by the immune system ✖ potential toxicity ✖ need for higher doses ✖ manufacturing and storage challenges
Dendrimers	✔ high water solubility and biocompatibility ✔ precise targeting of therapeutic nucleic acids ✔ enhanced drug bioavailability ✔ structural versatility ✔ ability to encapsulate various drugs	✖ potential toxicity ✖ complex and expensive synthesis ✖ lack of predictive tools for drug binding
Chemical conjugates	✔ enhanced cellular uptake ✔ enhanced bioavailability ✔ targeted delivery via lipid pathways ✔ potential for reduced side effects	✖ complex and expensive synthesis ✖ potential for off-target ✖ effects and immune reactions
Hydrogels	✔ controlled drug release ✔ efficient encapsulation ✔ high biocompatibility	✖ manufacturing challenges ✖ potential for premature drug release ✖ limited compatibility with hydrophobic drugs

**Table 2:** Methods of delivering nucleic acids inhibitors and vaccines against influenza A, SARS-CoV-2, and RSV viruses presented in the paper

Type of therapeutic	Modification	Method of delivery	Experimental model	Target virus	Reference
ASO	PTO	CPP	A549 cells	IAV	Ding et al.^98^
ASO	LNA 2′OMe	Lipofectamine 2000	MDCK cells A549 cells	IAV	Markov et al.^99^; Lenartowicz et al.^100^; Michalak et al.^101^; Soszynska-Jozwiak et al.^102^
siRNA	none	hybrid microcarriers	A549 cells	IAV	Brodskaia et al.^103^
siRNA	LNA 2′ -F PTO 2′OMe	Lipofectamine 2000	MDCK cells	IAV	Piasecka et al.^104^
mRNA	m1Ψ CAP-1	self-assembled ferritin	C57BL/6 mice	IAV	Di et al.^105^
mRNA	m1Ψ	LNP	FcγRIV mice; human	IAV	Freyn et al.^106^
ASO	PTO LNA	Lipofectamine 3000; JetPEI; intranasal instillation	MDCK cells Huh-7 cells ACE2-TMPRSS2-Huh-7.5 cells ACE2-A549 cells BALB/c mice Syrian hamster	IAV SARS-CoV-2	Hagey et al.^107^
mRNA	CAP-1	Lipofectamine MessengerMAX Reagent LNP	HEK293T cells BALB/c mice	SARS-CoV-2 IAV	Ye et al.^108^
mRNA	unknown	LNP	K18-hACE2 mice; human	SARS-CoV-2 IAV	Kreier et al.^109^
mRNA	m1Ψ CAP-1	LNP	K18-hACE2 mice; human	SARS-CoV-2	Meo et al.^110^; Food and Drug Administration^111^^,^^112^^,^^113^
siRNA	2′OMe	Lipofectamine 2000; LNP	VeroE6 K18-hACE2	SARS-CoV-2	Idris et al.^114^
ASO	LNA	Lipofectamine 3000; intranasal instillation	Huh-7 cells K18-hACE2 mice	SARS-CoV-2	Zhu et al.^115^
siRNA	2′OMe 2′-F PMO PTO	Lipofectamine RNAiMAX intranasal instillation intratracheally injection	A549 cells BALB/c mice FVB mice	SARS-CoV-2	Hariharan et al.^116^
siRNA, ASO gapmers	PTO 2′OMe LNA	Lipofectamine 3000	HEK293T cells	SARS-CoV-2	Baliga-Gil et al.^117^
siRNA	2′OMe PTO 2′-F	Lipofectamine RNAiMAX intranasal instillation aerosol inhalation	Vero E6 cells K18-hACE2 mice	SARS-CoV-2	Chang et al.^118^
siRNA	2′OMe PTO	Lipofectamine RNAiMAX	Vero E6 cells	SARS-CoV-2	Tolksdorf et al.^119^
siRNA	2′OMe PTO	Lipofectamine 2000 LNP	Vero E6 cells; A549 cells K18-hACE2 mice	SARS-CoV-2 RSV	Supramaniam et al.^120^
siRNA	none	Lipofectamine 2000 intranasal instillation	Vero E6 cells BALB/c mice	RSV	Alvarez et al.^121^
siRNA	none	intranasal instillation	BALB/c mice	RSV	Bitko et al.^11^
ASO	LNA	Lipofectamine RNAiMAX	A549 cells	RSV	Wu et al.^122^
mRNA	m1Ψ CAP-1	LNP	BALB/c mice	RSV	Espeseth et al.^123^

Abbreviation of nucleic acid modifications: 2′-OMe, 2′-O-methyl RNA; 2′-F, 2′-fluoro RNA; LNA, locked nucleic acid; PTO, phosphorothioate oligonucleotide; PMO, phosphorodiamidate morpholino oligomer; m1Ψ, N1-methylpseudouridine; CAP 1, CAP structure on 5′ end of mRNA: m7GpppNm, where Nm is a 2′-methylated nucleotide.

### Naked oligonucleotides

Numerous studies have shown that the systemic administration of naked oligonucleotides via the bloodstream is constrained by their instability and suboptimal pharmacokinetic properties. Naked oligonucleotides are highly vulnerable to degradation by nucleases present in the body, resulting in a shortened half-life and diminished therapeutic efficacy. Furthermore, their limited capacity to penetrate cell membranes without the assistance of a delivery vehicle significantly restricts their effectiveness.^124^ Nevertheless, local pulmonary delivery of modified naked oligonucleotides has shown promising results in treating lung infections.^125^ The application of chemical modifications enhances the stability, bioavailability, and specificity of naked oligonucleotides. By altering the chemical structure, such as through modifications to the 5′ and 3′ ends or the substitution of phosphate groups, oligonucleotides can be safeguarded from enzymatic degradation, while also improving their ability to penetrate cell membranes.^126^ This is particularly significant as unmodified oligonucleotides often necessitate frequent administration due to their rapid clearance from the bloodstream.^65^ The intranasal administration of modified, specific oligonucleotides may reduce the required dose for treating lung diseases compared to oral administration methods.^127^ Furthermore, unlike systemic administration, which increases the risk of degradation of siRNA or ASOs by serum proteins, direct delivery to the lungs circumvents such interactions, as serum proteins do not interact with the lung mucosa.^128^ This targeted delivery approach helps address some of the challenges associated with the systemic use of unmodified oligonucleotides while simultaneously capitalizing on their advantages, such as low immunogenicity and ease of synthesis.^129^ Systemic administration of naked oligonucleotides, while enabling extensive tissue distribution, is considerably constrained by rapid enzymatic degradation, limited cellular uptake, and potential interactions with serum proteins. These factors necessitate frequent dosing or chemical modifications to maintain therapeutic efficacy. In contrast, local delivery methods, such as pulmonary or intranasal administration, offer enhanced stability and bioavailability by circumventing systemic circulation, thereby reducing enzymatic degradation and lowering the required therapeutic dose. However, this approach is limited to target tissues that are accessible, and effective cellular uptake may still depend on the development of optimized delivery systems. Despite these limitations, the low immunogenicity and ease of synthesis position naked oligonucleotides as a promising platform for targeted therapeutic applications.^124^^,^^126^

### Nanoparticles

#### Lipid and lipid-based nanoparticles

RNA-based therapeutic nucleic acids are hydrophilic molecules that carry a negative charge. As a result, the development of efficient cellular delivery methods poses a significant challenge, one approach to which involves the use of nanoparticles. A notable example of such nanoparticles is lipid nanoparticles (LNPs). The standard composition of LNPs includes ionizable lipids, cholesterol, helper lipids, and polyethylene glycol (PEG)-grafted lipids. PEG forms a hydration shell that acts as a protective barrier, preventing the therapeutic nucleic acids from interacting with biological macromolecules other than those specifically targeted by the treatment.^130^ Among these components, ionizable lipids play a crucial role, as they undergo protonation upon endocytosis of the LNPs due to the reduction in pH.^131^ As a result, lipids acquire a positive charge, allowing them to interact with the negatively charged phosphate groups of nucleic acids. Consequently, LNPs can adhere to the surface of target cells and subsequently internalize. Ionizable lipids interact with the negatively charged components of the endosomal membrane, leading to its destabilization and promoting the release of nucleic acids into the cytoplasm.^132^^,^^133^ This procedure enhances delivery efficiency and reduces toxicity, an effect that is not observed in particles that do not undergo changes in their cationic charge at physiological pH. Lipids are also capable of self-organizing into well-ordered nanoparticle structures, referred to as lipoplexes, which arise from a combination of electrostatic interactions with nucleic acids and hydrophobic interactions.^134^ The efficiency of endosomal escape of RNA from the engineered nanoparticles is primarily determined by the structural characteristics of the ionizable lipids.^134^ Due to their stability, biocompatibility, and high encapsulation capacity, LNPs are the most commonly utilized nanoparticles for the delivery of mRNA vaccines.^135^ A key advantage of liposomes is their ability to protect therapeutic nucleic acids from external environmental factors, facilitate targeted delivery to specific tissues, and release their cargo in a controlled manner. Furthermore, their large core provides ample space for long RNA, shielding them from degradation until they reach the target cells. In addition to their biocompatibility and efficient RNA encapsulation, liposomes offer ease of preparation and modification, further enhancing their applicability in drug delivery.^131^ Despite their numerous advantages, LNPs also have certain limitations. One of the primary challenges is the method of their production, particularly the high-pressure homogenization (HPH) technique, which involves high operating temperatures and the application of cavitation forces that may affect the stability and quality of the encapsulated therapeutic nucleic acids. Additionally, LNPs must be tailored to encapsulate various therapeutic nucleic acids with differing physicochemical properties, which can lead to issues related to their stability and solubility, posing a significant technological challenge.^136^

#### Polymer and polymer-based nanoparticles

Innovative polymeric nanoparticles have been engineered for the encapsulation and delivery of nucleic acids within cationic polyplexes. Commonly utilized polymers in this process include poly(ethyleneimine) (PEI), poly(L-lysine), and protamine.^137^ Polymeric materials enable the formation of diverse nanoparticle structures and morphologies. The efficacy of the formulated nanoparticles is largely influenced by the specific combinations of polymers utilized, allowing for precise control over their properties and functionalities. The most commonly employed polymer-based materials include polysaccharides, chitosan, chitin, polyamines (such as polyethylamine), poly(amino acids) (such as poly(L-lysine)), polyesters, and various other polymeric compounds.^138^^,^^139^ The encapsulation of oligonucleotides within polymer complexes is primarily facilitated through ionic interactions, as the polymer contains positively charged functional groups. In addition to this mechanism, oligonucleotides may also become entrapped within the pores of the polymer particles, where alternative interactions contribute to their retention. Furthermore, a high concentration of positive charges on the polymer enhances the endosomal escape of oligonucleotides, thereby improving their release efficiency.^139^ Polymeric and polymer-based nanoparticles constitute a promising platform for the delivery of therapeutic nucleic acids, attributed to their capacity for controlled drug release. Furthermore, these nanoparticles possess the significant advantage of enhancing the bioavailability of therapeutic compounds characterized by poor water solubility. In addition, polymeric and polymer-based nanoparticles offer protection against the potential degradation of therapeutic nucleic acids, thus improving their stability and efficacy. Nonetheless, these nanoparticles are accompanied by several critical limitations. A primary concern is their potential toxicity, which necessitates the meticulous selection of materials to ensure biocompatibility. Additionally, the production process is complex and costly, particularly when scaled to industrial levels. Another notable limitation pertains to their physicochemical stability. Polymeric and polymer-based nanoparticles are prone to aggregation or alterations in their properties during storage, underscoring the necessity for establishing appropriate storage conditions. Furthermore, a significant challenge remains in the absence of clear regulatory guidelines governing their commercialization, which may impede their widespread adoption in clinical applications.^140^

#### Polycation-based nanoparticles (polyplexes)

The utilization of polycation-based nanoparticles for siRNA delivery enhances its therapeutic efficacy. These nanoparticles comprise synthetic or natural cationic polymers or incorporate cationic forms in which siRNA is encapsulated, forming polyplexes. The application of such nanoparticles facilitates the overcoming of extracellular and intracellular barriers, promotes the cellular uptake of charged particles, and enables effective interactions with intracellular targets.^141^ Polyplexes are the main polycationic system for siRNA delivery, formed by the self-assembly of siRNA with polycations (synthetic or natural). The formation of particles is driven by ionic interactions between positively charged groups (cationic amines) and anionic phosphates present in RNA. Due to the presence of a positive charge on the surface of polyplexes, it is possible to capture the cell membrane and interact with it, while siRNA is protected from degradation by nucleases.^142^ To formulate polyplexes, a cationic polymer is used with an amount much larger than the amount of oligonucleotide, which allows the obtaining of particles with a positive charge on the surface.^143^ In addition, it is possible to make surface modifications to polyplexes to increase their chances of delivery to specific targets. An example of such surface modification includes the application of hydrophilic polymers, such as poly(ethylene glycol) (PEG), a process known as PEGylation, as well as modifications involving chitosan or cyclodextrins. Thus, when designing, it is possible to create multifunctional systems that are able to deliver the drug more efficiently. Polyplexes present both advantageous and challenging attributes as carriers for therapeutic nucleic acids. The principal advantage of polyplexes arises from their ability to form complexes with siRNA through electrostatic interactions, which is a straightforward process. Furthermore, polyplexes, particularly those derived from PEI, enhance therapeutic efficacy by facilitating endosomal escape. Their structural composition permits modifications aimed at optimizing intracellular transport and enabling controlled release of therapeutic nucleic acids. Nevertheless, the clinical application of polyplexes is constrained by concerns related to cytotoxicity, particularly associated with high-molecular-weight polycations. Additionally, polyplexes tend to experience rapid clearance from the circulation, which diminishes their bioavailability; this phenomenon often necessitates modifications such as PEGylation to improve their pharmacokinetic profile. Moreover, the efficiency of delivery to target cells remains suboptimal, indicating a need for further structural refinements. Future prospects point to combining polyplexes with nanoparticles to reduce or prevent the immune response resulting from recognition of “foreign” siRNA. Despite these challenges, polyplexes continue to be regarded as promising carriers for therapeutic nucleic acids.^144^

### Liposomes

#### Cationic liposomes

Due to electrostatic interactions and the negative charge of oligonucleotides, they can be effectively encapsulated within cationic liposomes. Cationic liposomes consist of a cationic head group and a hydrophobic tail. The cationic head plays a crucial role in interacting with therapeutic nucleic acids.^145^ The functional components of cationic liposomes are cationic lipids, which can be classified as either monovalent or multivalent. The monovalent cationic lipid group includes compounds such as 1,2-dioleoyl-3-dimethylammonium propane (DODMA) and 1,2-dioleoyl-3-trimethylammonium-propane (DOTAP). Notably, cationic liposomes incorporating multivalent cationic lipids have demonstrated enhanced transfection efficiency for therapeutic nucleic acids.^146^^,^^147^ A widely used commercial transfection reagent based on cationic liposomes is Lipofectamine. This reagent is commonly employed for delivering nucleic acids into cells due to its high transfection efficiency and relatively low cytotoxicity. The primary advantage of cationic liposomes is their ease of production, which facilitates their application in clinical practice. Additionally, they exhibit low immunogenicity and possess the capability to form complexes with large fragments of therapeutic nucleic acids, thus enabling effective delivery to target cells. However, the use of cationic liposomes is accompanied by certain challenges, such as interactions with serum proteins that lead to the formation of a so-called “protein corona,” which may alter their biological properties. Another limitation is the reduced efficacy of delivery in *in vivo* conditions, as well as the potential for toxicity, which can impact their therapeutic application. Notwithstanding these limitations, cationic liposomes represent a promising alternative to viral vectors in gene therapy. Nonetheless, further research is necessary to overcome existing barriers and optimize their clinical application.^148^

#### Ionizable liposomes

Ionizable liposomes are composed of aminolipids containing amino groups that can undergo ionization. When designing ionizable liposomes, a critical factor to consider is the pKa value of the amine group, as it influences interactions with cell membranes and serum proteins. Additionally, the pKa value plays a key role in determining the potential toxicity of ionizable liposomes and their delivery efficiency.^149^ Ionized liposomes undergo protonation or deprotonation in response to environmental changes. Cargo-loaded liposomes initially exhibit a positive charge upon delivery; however, this charge shifts immediately before cellular administration. Following cellular uptake, liposomes acquire a negative charge, but upon cargo release, they regain a positive charge.^150^ Ionizable liposomes have the capability to effectively transport active substances directly to infected cells, thereby enhancing the efficacy of therapeutic interventions. One of the principal benefits of ionizable liposomes is their high biocompatibility and versatility. At physiological pH, ionizable lipids maintain a neutral charge, which minimizes toxicity and enhances the body’s tolerance to the administered therapeutic nucleic acids. Consequently, this contributes to a reduction in the risk of adverse effects, such as allergic reactions and tissue irritation. Another noteworthy advantage is their ability to facilitate the efficient release of therapeutic nucleic acids within target cells. Through the destabilization of the endosomal membrane induced by protonation, therapeutic nucleic acids can be readily released into the cytoplasm.^151^ Ionized liposomes do not have drawbacks that influence their efficacy and safety in application. However, despite their numerous advantages, the technology is not without limitations that may constrain its clinical application. One of the primary limitations is the potential for interaction between ionized liposomes and the immune system. Ionized liposomes undergo opsonization, resulting in their more rapid uptake by macrophages and subsequent elimination. This effect leads to a reduction in the duration of action of therapeutic nucleic acids, necessitating the use of higher dosages, which may, in turn, result in the occurrence of adverse effects. Additionally, another limitation pertains to the potential toxicity of ionized liposomes, as they may induce apoptosis and trigger pro-inflammatory responses. Furthermore, the production and establishment of storage conditions for ionized liposomes continue to pose significant technological challenges.^151^^,^^152^

### Dendrimers

Dendrimers are a class of polymeric macromolecules characterized by a well-defined structure consisting of a central core and highly branched, repeating units terminating in functional groups.^153^ Dendrimers have garnered significant attention due to their high water solubility, biocompatibility, and well-defined molecular weight. These characteristics make them highly suitable as carriers for drug delivery, and they have been demonstrated to be effective in facilitating the cellular transport of siRNA and mRNA.^154^^,^^155^ The poly(amidoamine) (PAMAM) dendrimer is the most commonly used dendrimer for therapeutic delivery. The localization of a therapeutic nucleic acids within the dendrimer is determined by both the dendrimer’s structural characteristics and the physicochemical properties of the therapeutic compound. A critical aspect of the drug encapsulation process is the careful design or selection of an appropriate dendrimer. At present, no reliable predictive tools exist to assess the binding affinity of dendrimers for specific drugs. Consequently, to identify optimal dendrimer-drug combinations, systematic screening studies involving various dendrimers are recommended.^156^ Additionally, a vaccine based on dendrimer nanoparticles (MDNPs) has been successfully developed. Preclinical studies conducted in mice demonstrated that a single intramuscular dose effectively induced immunity against H1N1 influenza virus infections, as well as Ebola virus and *Toxoplasma gondii*.^157^^,^^158^ Dendrimers are highly branched polymers that are employed in drug delivery systems, including the administration of antiviral medications for the treatment of respiratory diseases. Their distinctive architecture enables the precise targeting of active pharmaceutical ingredients to specific cells, thereby minimizing adverse effects on healthy tissues and reducing the incidence of side effects. Furthermore, dendrimers enhance drug bioavailability and facilitate the translocation of therapeutic nucleic acids across biological barriers. Due to the presence of a multitude of functional groups on their surfaces, these polymers can be modified to tailor their properties to meet specific therapeutic requirements. Notwithstanding their numerous advantages, dendrimers also present certain limitations. In particular, those of higher generations may exhibit toxic properties, thereby restricting their clinical applicability. The synthesis of dendrimers is both complex and expensive, which poses significant challenges for large-scale production. Additionally, it is noteworthy that not all dendrimers possess adequate biodegradability, which may result in their accumulation within the body and potentially lead to adverse health effects.^159^^,^^160^

### Chemical conjugates

Most conjugation strategies are primarily designed to improve biochemical properties and enhance cellular uptake efficiency. Cell-penetrating peptides (CPPs) are widely utilized for nucleic acid delivery due to their ability to traverse cell membranes. Although the precise mechanism underlying CPP interaction with the cell membrane remains incompletely understood, it is hypothesized that CPPs facilitate the clustering of negatively charged glycosaminoglycans on the cell surface. This clustering is thought to induce macropinocytosis and lateral diffusion or directly compromise the integrity of the lipid bilayer.^161^ The advantage of employing CPPs lies in the ability to deliver large therapeutic nucleic acids without the need for additional carriers. However, the challenges associated with the precise design of CPPs to selectively target infected cells present a significant hurdle. Consequently, further research is required to optimize their structure and mechanism of action.^162^

Another frequently used chemical conjugate is cholesterol. Attaching hydrophobic compounds to ASOs results in improved delivery *in vitro* by promoting release from endosomes. Such modification improves the ability of oligonucleotides to actively target specific sites in the body by exploiting natural lipid transport pathways.^163^ A similar conjugation unit to cholesterol is a fatty acid. This hydrophobic conjugate perfectly mimics the phospholipid bilayer and facilitates the introduction of oligonucleotides conjugated to it. Fatty acid can serve as an optimal conjugate for the delivery of therapeutic nucleic acids into muscle.^164^ The advantage of the conjugates in question is their ability to improve the solubility and bioavailability of drugs, which can lead to more effective therapeutic effects. Using natural carriers like high-density lipoprotein (HDL) and low-density lipoprotein (LDL) allows for selective drug delivery, potentially minimizing side effects. However, the process of synthesizing stable and effective conjugates can be complex and expensive. It is also important to keep in mind that conjugates may exhibit undesirable interactions with other drugs or lead to unforeseen immune reactions.^165^

α-tocopherol is an isomer of vitamin E and belongs to the group of fat-soluble compounds. It is an essential ingredient in the diet, making it an ideal candidate for a non-toxic and safe chemical conjugate. Due to its structure, containing a chromate group with a hydroxyl group and a hydrophobic saturated side chain, it can potentially increase membrane permeability. These properties make it a suitable conjugate for the delivery of siRNA and ASOs.^166^ However, its lipophilic nature may limit its solubility in aqueous environments, which is an obstacle to effective drug delivery to target sites in the body.^167^‬‬‬‬‬‬‬‬‬‬‬‬‬‬‬‬‬‬‬‬‬‬‬‬‬‬‬‬‬‬‬‬‬‬

Triterpene molecules can also be used as chemical bioconjugates. Squalene is an example of such a molecule. Thanks to its hydrophobic nature, it can be a suitable candidate for an oligonucleotide conjugate. The combination of oligonucleotides with squalene in an aqueous environment leads to the formation of non-toxic, self-organized nanoparticles. Squalene is used to improve the hydrophobicity and stability of oligonucleotides, which leads to a positive outcome of therapeutic delivery.^168^ An advantage of triterpenes is their ability to inhibit various stages of the viral life cycle. Conjugates of echinocystic acid with galactose show effectiveness in inhibiting the entry of IAV into Madin-Darby canine kidney (MDCK) cells by binding to the virus’ hemagglutinin, which interferes with its interaction with sialic acid present on the surface of host cells. In addition, triterpenes have increased water solubility and reduced hemolytic activity, making them safer and more effective as potential antiviral agents. One of the main limitations in the use of triterpenes is their low water solubility, which significantly hinders their bioavailability and limits their therapeutic efficacy. In addition, their high polarity can adversely affect permeability across biological membranes, which is important for reaching adequate concentrations at target sites.^169^

Another example of a bioconjugate is N-acetyl galactosamine (GalNAc). This monosaccharide binds with high specificity and affinity to the asialoglycoprotein receptor (AGPR) that is expressed in the liver. The conjugation of GalNAc with oligonucleotides and siRNA enabled the efficient delivery of these molecules to hepatocytes.^150^^,^^163^ The utilization of GalNAc conjugates presents a significant advantage in the precise delivery of therapeutic nucleic acids to target hepatocytes, thereby minimizing side effects and enhancing treatment efficacy. Moreover, GalNAc is characterized by its notable stability and biocompatibility, rendering it an appealing carrier for nucleic-acid-based therapies. However, its specificity toward ASGPR receptors results in challenges in the effective delivery of therapeutics to respiratory cells. Although GalNAc facilitates efficient and selective drug delivery to the liver, its application in treating respiratory infections remains constrained due to its limited affinity for respiratory cells.^170^^,^^171^

Other examples of receptor conjugates, besides GalNAc, are antibodies and aptamers. The most commonly used antibodies are monoclonal antibodies. They have found use as a means of delivering oligonucleotides to cells. Antibodies can be used in the form of direct conjugates and uncoupled carriers.^172^ An example of an application is an antibody specific to the transferrin receptor. This receptor is involved in the intracellular transport of iron-bound transferrin. The use of this antibody in combination with siRNA allows its transport into muscle tissue.^173^ The primary advantage of employing antibodies lies in their ability to precisely deliver therapeutics to infected cells. Due to their specificity, antibodies can guide highly toxic drugs directly to target cells, thereby enhancing the effectiveness of therapy and reducing side effects.^174^ However, antibodies face inherent limitations in penetrating cell membranes, which complicates their access to internal cellular structures. Another challenge is ensuring the stability and uniformity of the antibodies. Furthermore, there exists a risk of immunogenicity associated with the introduced antibodies, which can compromise their efficacy and lead to adverse effects.^174^^,^^175^

Aptamers are single-stranded oligonucleotides with a length of 20–100 nt. Similar to antibodies, aptamers have an affinity for receptors.^176^ Aptamers show the ability to bind specifically to disease biomarkers. Due to their favorable properties, they are considered promising in developing aptamer-therapeutic systems. Aptamers are characterized by rapid penetration into tissues, ease of formulation, and relatively low immunogenicity, making them suitable candidates for use as therapeutic conjugates.^177^ The ability of aptamers to selectively bind to target molecules, which stems from their unique three-dimensional structure, constitutes a significant advantage. In contrast to traditional antibodies, aptamers are characterized by their smaller size, high specificity and affinity, favorable biocompatibility, robust stability, and low immunogenicity, rendering them particularly appealing for biomedical applications.^178^ However, despite these advantages, certain challenges are associated with the utilization of aptamers in the delivery of therapeutic nucleic acids. One of the primary issues is their susceptibility to degradation by nucleases present in serum, which may reduce their therapeutic efficacy within the body. Furthermore, chemical modifications intended to enhance the stability of aptamers may lead to undesirable outcomes, including hepatotoxicity and the induction of immune responses.^178^

### Hydrogels

Hydrogels are cationic carriers based on polymers capable of forming complexes with nucleic acids. Separate administration of siRNA and hydrogel causes their aggregation with proteins, but administration of siRNA-hydrogel complex no longer shows this activity, which is a promising method of siRNA delivery. Nanohydrogels are obtained by polymerization of pentafluorophenyl methacrylate and tri(ethylene glycol)-methyl ether methacrylate.^179^ Therapeutic nucleic acids can be introduced into the hydrogel in two forms: as naked nucleic acid or encapsulated in a nanocarrier. However, the introduction of naked oligonucleotides into the carrier is dependent on the interactions between the oligonucleotides and the hydrogel network. The characteristics that describe hydrogels are biocompatibility, biodegradability, and controlled drug release, which are a consequence of their three-dimensional backbone.^180^ Hydrogels represent a promising drug delivery system, particularly for therapeutic antiviral agents targeting respiratory viruses. Their biocompatibility and the ability to tailor physicochemical properties facilitate controlled drug release, which may enhance therapeutic efficacy while minimizing adverse effects. However, hydrogels also exhibit certain limitations. Generally, their hydrophilic nature may impede the effective incorporation and controlled release of hydrophobic antiviral drugs, presenting a challenge in treating numerous viral infections of the respiratory system. Furthermore, the poor mechanical strength of some hydrogels may result in premature drug release prior to reaching the intended site of action, thereby reducing the overall effectiveness of the therapy. Additionally, certain hydrogels may exhibit limited biodegradability, which could lead to their accumulation within biological systems and potential immunological reactions. The complexity of the production process and the necessity of precisely tailoring the properties of hydrogels to meet specific therapeutic requirements also present significant challenges in their application as delivery systems for antiviral medications.^160^^,^^181^

## Delivery approaches for therapeutic nucleic acids against respiratory RNA viruses

Ding et al. used phosphorothioate-bond-modified ASOs to interfere with the vRNA replication of IAV subtype H1N1 using delivery vehicle CPP. ASOs were delivered to A549 cells infected with this virus; then, the approach was tested on mouse models. For this purpose, HABP (hemagglutinin-binding peptide) ligands and the fluorescein (FAM) were attached to ASOs. The modified ASOs were efficiently introduced into the infected cells by binding to viral HA proteins, resulting in the inhibition of IAV replication.^98^

Another study, also, using ASOs was conducted by Markov et al. The ASOs tested were designed to target mRNAs and genome RNA segments encoding subunits of the polymerase complex PB1, PB2, and PA of IAV. Moreover, ASOs were modified by incorporation LNA and 2′-O-methyl groups. The study used Lipofectamine 2000 to introduce ASOs into MDCK and A549 cells. The experiment resulted in a 15-times reduction in virus titer in the infected cells for the best ASOs, compared to the control cells.^99^

An effective method for delivering a mixture of different naked siRNAs targeting conserved IAV structural motifs was described by Brodskaia et al. For this purpose, hybrid encapsulation microcarriers containing therapeutic siRNA targeting the NP, NS, and PA protein-coding regions were used. The hybrid microcarriers are composed of biodegradable polymers and SiO_2_. These nanostructures are characterized by low toxicity to cells in *in vitro* conditions and effectively protect siRNA from degradation by RNases. Delivered in such a way, siRNAs to A549 cells caused replication inhibition of various IAV subtypes (including H1N1). The authors, therefore, showed a promising vehicle system that exhibits low cytotoxicity and high delivery efficiency.^103^

The study by Piasecka et al. used various modified siRNAs (substituted with LNA, DNA, 2′-F, 2′-O-methyl, and phosphorothioate nucleotide residues) targeting the structural motifs of the nucleoprotein (NP) mRNA of the IAV. For this purpose, Lipofectamine 2000 was used to introduce the therapeutic RNAs into MDCK cells. The experiment resulted in a 90% efficiency of inhibition of IAV replication by the best designed siRNAs. The results indicate that conserved structural motifs can serve as effective targets for siRNA.^104^

A design approach based on the determined secondary structure of viral genomic RNA was applied to evaluate ASOs targeting segment 8 of IAV. MDCK cells were transfected with ASOs using Lipofectamine 2000. The most effective ASOs targeted weakly-paired and single-stranded regions as hairpin and internal loops, confirming that the effectiveness of ASOs is influenced by the structure and accessibility of RNA. It is worth noting that the incorporation of chemical modifications (2′-O-methyl and LNA) to the ASOs has increased their efficacy.^100^

In addition, Michalak et al. and Soszynska-Jozwiak et al. designed ASOs targeting segment 5 RNA, both (−) and (+) strands, of the IAV. All oligonucleotides were 2′-O-methyl-RNA, and several had LNA modifications. MDCK cells were transfected with Lipofectamine 2000. The most potent oligonucleotides reduced viral titers by ∼90% and 87%, targeting (−) and (+) segment 5, respectively.^101^^,^^102^

Hagey et al. proposed the therapeutic targeting of a conserved secondary structure motif of IAV RNA, packaging stem-loop 2 (PSL2), with an analogous approach for SARS-CoV-2. PSL2 is a conserved secondary structure of the IAV PB2 vRNA. According to the predictions of the authors, this region is less prone to mutation in response to targeted therapeutics. This structure is indirectly involved in viral RNA packaging *in vitro* and is present in all known IAV strains. MDCK cells were used for IAV, Huh-7, ACE2-TMPRSS2-Huh-7.5, and ACE2-A549 lines for SARS-CoV-2. Animal studies included BALB/c mice for IAV and Syrian hamsters for SARS-CoV-2. Nine variants of modified ASO gapmers with LNA and DNA core that activate RNase-H to degrade PSL2 were created. The LNA-ASOs, containing phosphorothioate backbone, were delivered to the animals by the intranasal route using a mixture of Lipofectamine 3000 and JetPEI (transfection reagent [Polyplus]). The results showed 100% survival in mice and a strong immune response, whereas prophylactic administration of LNA-ASO protected hamsters from SARS-CoV-2 USA_WA1/2020 transmission. LNA-ASOs designed to disrupt the structure of PSL2 dramatically inhibit IAV *in vitro* against viruses of different strains and subtypes.^107^

Several publications showed the application of siRNA targeting SARS-CoV-2 RNA using selected delivery. Knowledge of the highly conserved motifs of SARS-CoV-2 RNA contributed to attempts to use therapeutic siRNAs targeting these motifs. The experiment by Idris et al. used motifs located in the RdRp, helicase regions and the 5′ UTR. Multiple modified (2′-O-methyl, PTO nucleotide residues) siRNA variants were tested, and three inhibited viral replication with 90% efficiency, applied individually or in combination with each other. Two LNPs were developed and tested for their delivery to mouse models, and the study showed that the delivered siRNAs alone or in combination with each other showed the same inhibition of the virus, regardless of the concentration of the individual siRNAs. It was shown that the LNPs used could be applied to deliver therapeutic siRNAs. However, RNAi-based technologies and LNPs are still new and need to be refined. However, the approach appeared to be relatively safe and promising in targeting SARS-CoV-2.^114^

Zhu et al. designed multiple LNA ASOs targeting the critical SL1 structure in the 5′ leader sequence of SARS-CoV-2. SL1 is a highly conserved structure in the 5′ UTR of the SARS-CoV-2 genome, playing a key role in the initiation of viral RNA translation, which makes it an attractive therapeutic target, as its disruption can effectively inhibit viral replication. In the first stage of the study, they tested the effectiveness of ASOs by infecting Huh-7 human hepatoma cells with the native virus. ASOs were delivered into the cells using Lipofectamine 3000. Cellular studies have selected the oligonucleotide that most efficiently reduces viral replication. Next, they investigated the antiviral effects of ASOs by employing humanized transgenic K18-hACE2 mice. Mice were administered intranasally with a potential inhibitor dissolved in saline. They demonstrated that both ASO-treated cells and mice showed a significant decrease in viral replication.^115^

To assess the potential of designed siRNAs and ASO gapmers to modulate SARS-CoV-2 virus infection, Hariharan et al. modified A549 cells to overexpress human angiotensin-converting enzyme 2 (ACE2) and TMPRSS2. Modified siRNAs (2′-O-methyl, 2′-fluoro groups, phosphorothioate, and phosphodiester backbone) were delivered to the cells by Lipofectamine RNAiMAX. The most effective oligonucleotides reducing viral mRNA level by 99% targeted genomic regions: ORF7a and nucleocapsid. In the next step, the selected siRNAs were applied with intranasal instillation or intratracheal injection into BALB/c or FVB mice. They noted a reduction of over 10-fold in lung viral titers and a 25- to 100-fold reduction in viral RNA levels.^116^

Another example of the use of ASOs to inhibit the SARS-CoV-2 replication cycle was described by Baliga-Gil et al. The ASO gapmers and siRNAs targeting sgRNA N of SARS-CoV-2 were used. All ASOs had 2′-O-methyl, LNA modifications and phosphorothioate backbone to improve cellular properties and stabilize binding to targets. HEK293T cells were transfected with eukaryotic expression vectors encoding SARS-CoV-2 sgRNA N and oligonucleotides using Lipofectamine 3000. The experiment showed specific inhibition of the translation of N protein.^117^

After transfection using Lipofectamine RNAiMAX of unmodified siRNAs targeting coding region of SARS-CoV-2 RdRp into Vero E6 cells, Chang et al. selected the most effective ones. Then, the authors fully modified them by 2′-O-methyl, 2′-fluoro and phosphorothioate substitution for nuclease protection. To determine the antiviral activity, an animal model—K18-hACE2 transgenic mice—was used. Intranasal instillation and aerosol inhalation were used to deliver siRNAs to the lungs of SARS-CoV-2-infected mice. RT-qPCR shows a reduction of viral RNA by 96.2%. Moreover, the distribution of modified siRNA delivered via aerosol inhalation was uniform throughout the entire lung, unlike intranasal instillation delivery. However, quantitative analysis showed that intranasal instillation had a significantly higher dosing efficiency in the nasal cavity.^118^

Tolksdorf et al. developed modified siRNAs directed against the conserved 5′-UTR leader sequence of SARS-CoV-2. The 2′-O-methylase and phosphorothioate modifications resulted in increased stability and resistance to degradation. siRNAs were delivered into the cells using Lipofectamine RNAiMAX where they effectively inhibited viral replication, reducing virus level by two orders of magnitude and preventing cytopathic effects.^119^

The conducted experiment using siRNA (ALN-RSV01) targeting the N gene of the RSV nucleocapsid demonstrated its ability to inhibit virus replication in infected cells. The experiment used Vero E6 cells cultured in DMEM medium. Selected siRNA was delivered to the cells via Lipofectamine 2000. Next, Alvarez et al. used the BALB/c mouse to evaluate the antiviral effect of ALN-RSV01. Dissolved in PBS, siRNA was administered intranasally. The effectiveness of the delivered siRNA was confirmed by identifying a specific RSV mRNA cleavage site, which is the basis for conducting further studies on the inhibition of RSV replication using ALN-RSV01.^121^

Therapeutic RNA delivery to cells using Lipofectamine 2000 against RSV and SARS-CoV-2 was also described by Supramaniam et al. The experiment was initially conducted on two cell lines, A549 and Vero E6, respectively, and then focused on a K18-hACE2 mouse model. The final results demonstrated the efficacy of LNP-encapsulated modified (2′-O-methyl and phosphorothioate backbone) siRNA delivery to counteract viral infections affecting the respiratory system.^120^

Bitko et al. have proposed unmodified siRNAs targeting mRNA of P protein, a significant subunit of RSV viral RNA-dependent RNA polymerase. The diluted in Opti-MEM naked siRNAs were applied intranasally into mice. The research has shown that mice infected with the RSV lost weight, whereas mice treated with siRNA gained weight without interruption. In addition, the determination of RSV titers in the lungs showed that mice infected with the virus and treated with siRNA had a ∼99.98% reduction in viral titer.^11^

Wu et al. present an investigation of the potential functional relevance of host mRNA-RSV interaction. They used designed LNA-ASO targeting KANK2 and CD44 mRNA to block specific RNA-RNA interactions. ASOs are delivered to A549 cells via Lipofectamine RNAiMIX. Measuring GFP level by flow cytometry shows repressed RSV replication by more than 60% concerning control.^122^

## Approved by EMA and FDA mRNA vaccines for respiratory viruses and their delivery approaches

Freyn et al.. presented studies using nucleoside-modified mRNA vaccines encapsulated in LNPs against IAV. Moreover, mRNA has N1-methylpseudouridine modification to prevent detection by RNA sensors, reducing excessive inflammation and promoting increased protein expression. In a mouse experimental model, the vaccines delivered combinations of antigens (HA, NA, M2, NP) and induced immune responses. As a result, the studies showed that nucleoside-modified mRNA-LNPs vaccines provided protection against IAV infection. These results indicate significant progress in developing nucleoside-modified mRNA-LNPs vaccines that allow for the expression of multiple conserved antigens, making them promising universal vaccine candidates against influenza virus.^106^

IAV vaccines based on the conservative HA2 and M2 epitopes have the potential to provide protection against new variants of the virus.^182^^,^^183^ There have been studies suggesting the use of a multi-epitope vaccine against H1N1 and type B influenza viruses. The work of Di et al. involved the development of a vaccine containing three conserved IAV antigens, including the extracellular domain of the M2 ion channel (M2e), an epitope derived from hemagglutinin H2 (for types H1, H3, and B), and hemagglutinin H1. The modified mRNA was altered by introducing m1Ψ and the CAP-1 structure. Influenza virus mRNA vaccines were delivered using self-assembled nanoparticles. Three forms of mRNA vaccines have been developed: a monomeric structure (MH), a trimeric structure (MH-T), and a ferritin-based construct (MH-TF). Immunization tests were carried out on C57BL/6 mice, demonstrating that the proposed vaccine induces both humoral and cellular responses of CD4+ and CD8+ T lymphocytes. The results obtained indicate an enhanced cross-protection against infection with H1N1 and B influenza viruses.^105^

The first FDA-approved mRNA vaccine against SARS-CoV-2 was Pfizer/BioNTech’s BNT162b2. The vaccine contains modified (m1Ψ, CAP-1) mRNA encoding the S-protein of the virus and was delivered via LNP into the body to induce an immune response. The delivered vaccine persists in the body for a short time and does not bind to the host’s genetic material. The efficacy of Pfizer/BioNTech’s BNT162b2 vaccine in preventing COVID-19 was initially estimated at 95% in clinical trials.^184^^,^^185^ Later studies indicate that the vaccine’s efficacy is now estimated at 91% after two doses.^110^^,^^111^^,^^186^ This vaccine has also received approval for use from the EMA.^28^ The FDA, on August 22, 2024, approved for emergency use of the Pfizer/BioNTech vaccine against COVID-19 (Formula 2024-2025) containing a single component corresponding to the Omicron KP.2 variant of the SARS-CoV-2 strain.^112^

The second FDA- and EMA-approved mRNA vaccine against SARS-CoV-2 is Moderna mRNA-1273. Like Pfizer/BioNTech’s BNT162b2 vaccine, it contains mRNA with m1Ψ and CAP-1 structure on the 5′ end, encoding glycoprotein S, and is delivered by LNP.^27^^,^^28^^,^^110^ The authors observed protection against SARS-CoV-2 in mice following vaccination with the upgraded Formula 2024-2025, which includes a single-component formulation targeting the Omicron KP.2 variant of the SARS-CoV-2 virus. The updated vaccine has been approved for administration to individuals 6 months through 11 years of age.^113^

Ye et al. have designed an mRNA vaccine delivered via LNP, which simultaneously protects from both SARS-CoV-2 and IAV. The mRNA vaccine contained a sequence encoding the HA antigen of the IAV (ARIAV) and the receptor-binding domain (RBD) of the SARS-CoV-2 S protein (ARCoV). The study was initially conducted on HEK293T cells and subsequently on mice. The results demonstrated that vaccinated mice developed an antigen-specific immune response, indicating simultaneous protection against SARS-CoV-2 and IAV infection. Due to the similar size of ARIAV and ARCoV preparations, this is a clear advantage for their use as a combination vaccine. Furthermore, the antibody titer was comparable to that of ARIAV and ARCoV administered separately, suggesting that the combination vaccines are equally effective in generating an immune response. Combined with the significant advantages of mRNA technology, such as the possibility of rapid production, high safety profile resulting from the lack of penetration into the cell nucleus, and the ability to induce both humoral and cellular immune responses effectively, further design of universal, combined, vaccines based on mRNA platforms plays a key role in the effective control of SARS-CoV-2 and IAV infections and future respiratory disease pandemics.^108^

Studies using mRNA vaccines expressing RSV’s F protein have also been performed. The modified (CAP-1, N1-methylpseudouridine) mRNA was encapsulated in LNP to facilitate delivery to cells and enhance the immune response. The first stage of the studies included tests on Expi293F cells, to which mRNA was introduced using the ExpiFectamine Kit. It was shown that mRNA-LNP vaccine induce strong immune responses of cells against F protein of RSV. The next stage of the studies was the introduction of vaccine to mice and cotton rats. A strong immune response was also observed in both animal models.^123^ The RSV vaccine received official approval from both the FDA and EMA in 2023, in accordance with the findings from extensive international clinical trials.^31^

mRNA vaccines are considered promising in the context of combating viral co-infections. Moderna has completed phase III clinical trials of an mRNA vaccine targeting SARS-CoV-2 and IAV. The company is currently seeking FDA approval to bring the vaccine to market. The company also plans to develop a vaccine targeting SARS-CoV-2, IAV, and RSV.^109^

## Summary and future perspective

The occurrence of epidemics and pandemics caused by respiratory viruses has prompted the development of effective antiviral drugs. The strategy to target viral RNA with oligonucleotides is promising, and there are examples of its effectiveness. Also, developing an mRNA vaccine has demonstrated the great potential of nucleic-acid-based tools to combat and control the spread of respiratory viruses. Despite the advantages of oligonucleotides as therapeutic nucleic acids—direct interaction with the genome (or mRNA), and thus action at an early stage of infection, the possibility of rapid adaptation to strains, and even personalized therapy—there are also problems to overcome. The main interrelated problems are possible toxicity of therapeutic nucleic acids and difficulties in delivery. Any short oligonucleotide can cause off-target effects or undesired protein binding, causing immunostimulatory activity. Inflammatory and immune responses may also be induced by the chosen therapeutic carrier as an unintended effect. The future of antiviral oligonucleotides and mRNA vaccines lies in breakthroughs in delivery technologies. Advanced LNPs, exosome-based carriers, and biodegradable polymers hold promise for enhanced biodistribution and cellular uptake. The inhalation route, utilizing nanoparticle aerosols or dry powder formulations, may revolutionize localized lung delivery, increasing therapeutic efficacy while minimizing systemic exposure. The crucial direction is the refinement of oligonucleotide chemistry to improve stability and minimize off-target effects. AI-driven design of RNA-targeting molecules and next-generation sequencing will accelerate the development of personalized and strain-specific therapeutics. In mRNA vaccines, innovations such as self-amplifying RNA (saRNA) and circular RNA (circRNA) could enable stronger and longer-lasting immune responses with lower doses. The future of nucleic acid therapeutics against respiratory viruses is not just about refining existing tools but about pioneering entirely new platforms that merge precision medicine with scalable, patient-friendly delivery systems.

## Acknowledgments

This work was supported by the National Science Centre grants UMO-2020/39/B/NZ1/03054 and UMO-2021/41/B/NZ1/03819 to E.K. This research was partly funded by the 10.13039/501100004442National Science Centre, Poland, grant numbers UMO-2020/39/B/NZ1/03054 and UMO-2021/41/B/NZ1/03819. For Open Access publishing, the authors have applied a CC-BY public copyright license to any Author Accepted Manuscript (AAM) version arising from this submission.

The authors thank Marta Soszynska-Jozwiak for reviewing the manuscript and providing valuable suggestions. Figures 1, 2, 3, and 4 were created with BioRender.com.

## Author contributions

K.M. and A.B.-G. performed the literature review and wrote the manuscript. A.B.-G., visualization. E.K. wrote the manuscript and supervised the process. All authors have read and agreed to the published version of the manuscript.

## Declaration of interests

The authors declare no competing interests.
